# Localized dissection of the sinus of Valsalva mimicking spontaneous coronary artery dissection

**DOI:** 10.1093/icvts/ivad091

**Published:** 2023-06-08

**Authors:** Norihisa Yuge, Susumu Manabe, Koichiro Sugimura, Hiroaki Shimokawa

**Affiliations:** Department of Cardiovascular Surgery, International University of Health and Welfare Narita Hospital, Narita, Japan; Department of Cardiovascular Surgery, International University of Health and Welfare Narita Hospital, Narita, Japan; Department of Cardiology, International University of Health and Welfare Narita Hospital, Narita, Japan; Graduate School, International University of Health and Welfare, Chiba, Japan

**Keywords:** Coronary artery dissection, Localized dissection, Sinus of Valsalva

## Abstract

A 56-year-old man, suspected of having ST-segment elevation myocardial infarction due to spontaneous coronary artery dissection, underwent emergency percutaneous coronary intervention. Although he had moderate aortic regurgitation with aortic root dilation and mild heart failure, it was controlled with medications. Two weeks after discharge, he was readmitted with severe heart failure due to severe aortic regurgitation and underwent an aortic root replacement. Intraoperative findings revealed that localized dissection of the sinus of Valsalva involved the right coronary artery, resulting in coronary artery dissection. In cases of spontaneous coronary artery dissection, attention should be paid to coronary artery dissection caused by localized aortic root dissection.

## INTRODUCTION

Spontaneous coronary artery dissection (SCAD) is thought to be caused by medial dissection or rupture of the vasa vasorum of the coronary artery [1]. Stanford type-A aortic dissection is also known to cause coronary artery dissection, which can be difficult to differentiate from SCAD. We present a case of incidentally identified coronary artery dissection caused by localized dissection of the sinus of Valsalva.

## CASE REPORT

A 56-year-old man was admitted with chest pain. Electrocardiography revealed ST-elevation in leads II, III and aVF. The cardiac enzyme level was also markedly elevated (troponin T, 86,260 pg/ml).

**Figure 1:**
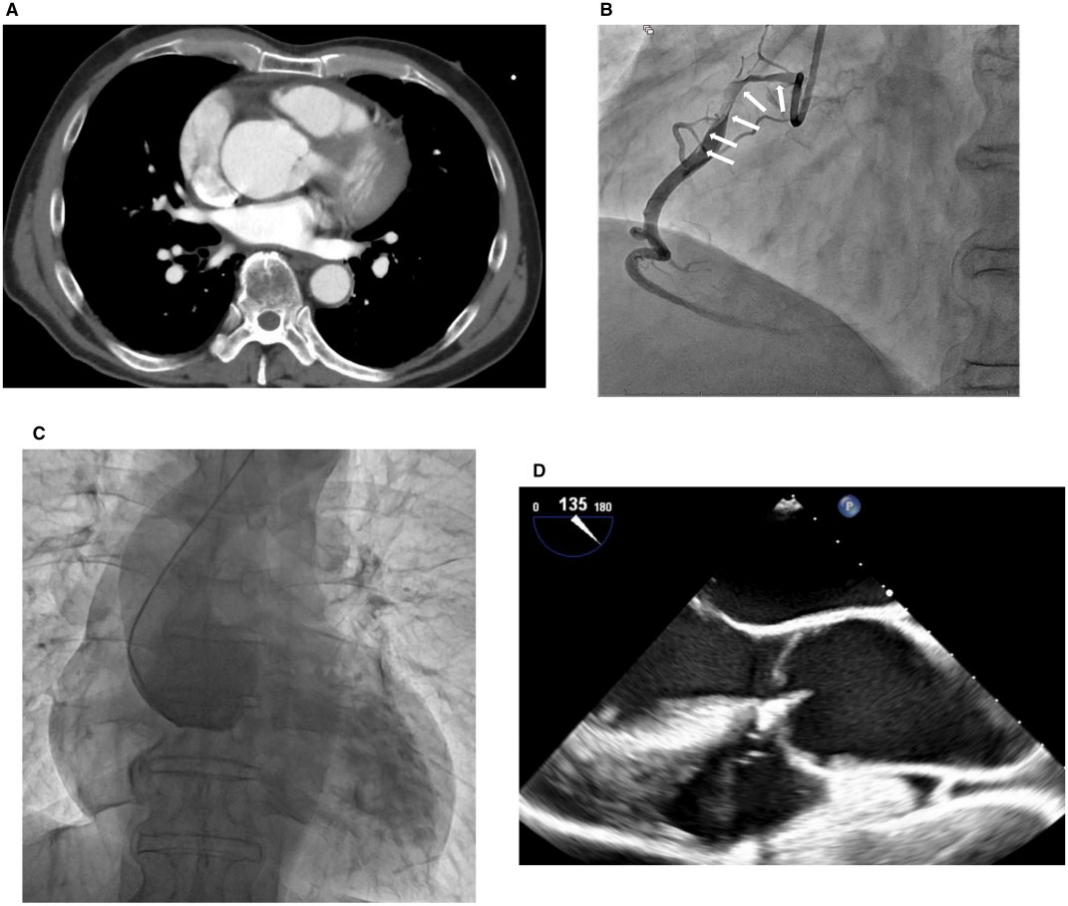
Enhanced computed tomography, coronary angiography and transoesophageal echocardiography findings. **(A)** Contrast-enhanced computed tomography images did not indicate an obvious intimal flap in the aortic root. **(B)** Coronary angiography revealing total occlusion of the right coronary ostium with an intimal tear (arrows). **(C)** Aortic root angiography revealing no flap. **(D)** Transoesophageal echocardiography revealing no intimal flap of the aortic root and the presence of prolapse of the non-coronary cusp.

Enhanced computed tomography (CT) showed dilation of the aortic root (sinus of Valsalva, 45 mm), but no dissection (Fig. 1A). Therefore, he was suspected of having ST-segment elevation myocardial infarction. Emergency coronary angiography revealed total occlusion of the right coronary ostium due to SCAD; percutaneous coronary intervention was performed (Fig. 1B). Aortic root angiography showed moderate aortic regurgitation (AR) but no intimal flap (Fig. 1C). Then, his chest pain was relieved. Because he had mild heart failure, he was treated with medications. He was discharged 20 days later.

After 2 weeks, he was readmitted with exacerbated dyspnoea at rest. Transthoracic echocardiography revealed severe AR with aortic root dilation. Consequently, we scheduled the patient to undergo an aortic root replacement operation. Preoperative transoesophageal echocardiography revealed no intimal tear of the aortic root, but he had a prolapsed non-coronary cusp (Fig. 1D).

**Figure 2.**
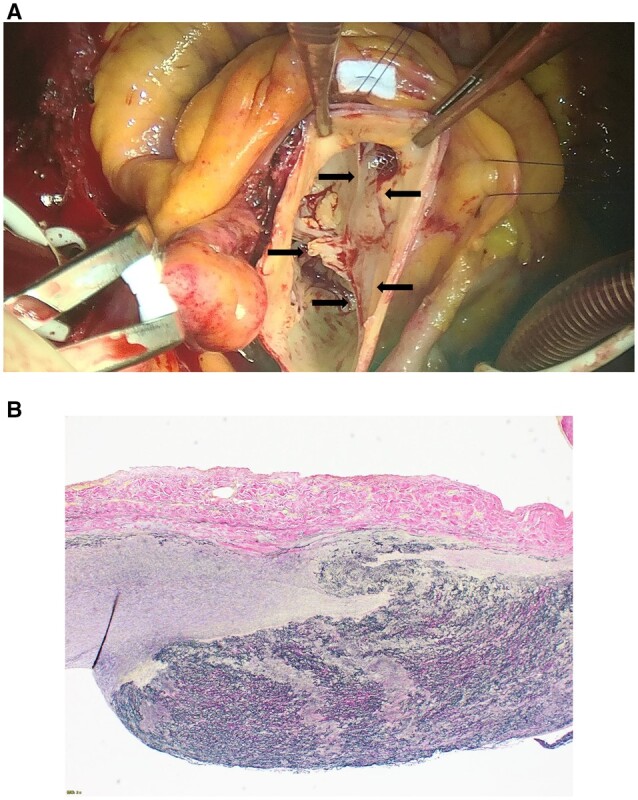
Intraoperative and histopathological findings. **(A)** An intraoperative photograph showing local dehiscence of the aortic commissure between the right and non-coronary cusps of the aortic valve (arrows). **(B)** Elastic van Gieson-stained section, showing a disruption of the media and a deficit of intima.

The operation was performed through a median sternotomy. The operative findings showed that the aortic root was dilated. The intima between the right and non-coronary cusps was ruptured, and the intimal defect extended into the right coronary ostium. We implanted a stent in the right coronary artery. Additionally, commissural dehiscence was observed between the right and non-coronary cusps that were prolapsed into the left ventricle, which was thought to be the cause of the AR. No intimal flap was observed (Fig. 2A). Then, we performed a Bentall procedure. The patient’s postoperative course was uneventful. Histological examination of the wall of the aortic root revealed disruption of the media and an intimal defect. Neointima was seen in the intimal defect, which indicated a chronic dissecting aortic aneurysm (Fig. 2B).

## DISCUSSION

SCAD occurs in less than 1% of patients with acute myocardial infarction [1]. More than 90% of the patients are women, and women under 50 years old and patients with a connective tissue disorder have an increased risk of SCAD. It is also more likely to occur in patients with low cardiovascular risk than in those with the factors related to typical acute coronary syndrome (ACS).

Aortic dissection is also known to cause coronary artery dissection [1]. Myocardial ischaemia due to Stanford type A acute aortic dissection (AAD) occurs in approximately 6% of patients and is considered a severe complication (mortality rate, 33%) [2]. In fact, a dissection extending into the aortic root may be misdiagnosed as ACS, and treatment for ACS may be initiated. Therefore, an accurate diagnosis is critical. Enhanced CT is useful in the diagnosis of AAD. However, unlike other common types of AAD, localized dissection of the sinus of Valsalva is difficult to diagnose due to artifacts such as cardiac motion and normal coronary cusps, which should be carefully assessed [3]. In the present case, CT at the onset of ST-segment elevation myocardial infarction revealed no obvious flap in the aortic root, and aortic dissection was revealed incidentally during the operation for AR. Some cases diagnosed as spontaneous coronary artery dissection may include cases in which coronary artery dissection is caused by a localized dissection of the sinus of Valsalva. Consequently, when coronary artery dissection is recognized, attention should be paid not only to coronary artery lesions but also to aortic root lesions.

**Conflict of interest:** none declared.

## Data Availability

The data underlying this article are available in Interdisciplivary CardioVascular and Thoracic Surgery, at https://doi.org/10.1093/icvts/ivad091.
